# Assessing Respiratory Tract Infections' Prevalence and Microbial Profiles in Mechanically Ventilated Patients: Insights From Broncho Alveolar Lavage Examination

**DOI:** 10.7759/cureus.58155

**Published:** 2024-04-12

**Authors:** Amit Anand, Kriti Maurya, Kaushik N R, Ranjith R, Chunawala Purvi Jatin, Ekta V Mallya, Sarosh Gilani, Afrin V S

**Affiliations:** 1 Anesthesiology and Critical Care, Autonomous State Medical College, Hardoi, IND; 2 Department of Microbiology, Autonomous State Medical College, Hardoi, IND; 3 Department of General Medicine, Rajiv Gandhi Government General Hospital, Chennai, IND; 4 Department of General Medicine, All India Institute of Medical Sciences, Rajkot, IND; 5 Department of General Medicine, New Vision University School of Medicine, Tbilisi, GEO; 6 Department of General Medicine, Bharati Vidyapeeth Deemed University, Pune, IND; 7 Department of General Medicine, North Eastern Indira Gandhi Regional Institute of Health and Medical Sciences, Shillong, IND

**Keywords:** american thoracic society for bronchoscopic lavage, respiratory infections, broncho alveolar lavage, mechanically ventilated patients, chest infection

## Abstract

Introduction

Chest infections represent a significant challenge in mechanically ventilated patients, often leading to adverse outcomes despite advancements in critical care. This prospective study was conducted in the intensive care unit of tertiary referral care, with objectives to assess chest infection prevalence, microbial profiles, and outcomes in mechanically ventilated patients through broncho-alveolar lavage (BAL) examination.

Methodology

This prospective study involved 38 patients aged 15 to 65 years who were receiving mechanical ventilation and underwent BAL. The procedure of BAL was followed as per the guidelines and recommendations outlined by the American Thoracic Society for Bronchoscopic Lavage. Microbial analysis involves the use of microscopic examination and quantitative culture methods. Different staining techniques were utilized to identify bacteria, fungi, and mycobacteria. Complications and adverse events were monitored and recorded.

Results

Out of the 38 patients who underwent BAL, the majority, 30 (78.94%), were found to have chest infections, with gram-negative bacteria, including Escherichia coli, Klebsiella pneumoniae, and Acinetobacter baumannii, being the causative agents. The antibiotic sensitivity profiles indicated that the organisms were susceptible to carbapenems and broad-spectrum β-lactam/β-lactamase inhibitor combinations while showing resistance to fluoroquinolones. Despite adequate treatment, mortality remained significant in 12 (31.57%) patients.

Conclusion

Study findings underscore the importance of proactive surveillance, early diagnosis, and targeted management strategies to mitigate the burden of respiratory infections in critical care settings.

## Introduction

Mechanical ventilation plays a crucial role in critical care, offering vital support to patients with respiratory failure due to various causes such as acute respiratory distress syndrome (ARDS), pneumonia, and exacerbations of chronic obstructive pulmonary disease (COPD) [1]. However, the use of mechanical ventilation is not without risks, and one of the most significant complications is the development of chest infections [2,3]. These infections, encompassing a spectrum from ventilator-associated pneumonia (VAP) to other forms of pneumonitis, represent a substantial burden on both patients and healthcare systems worldwide [4,5]. Despite advances in medical technology and critical care practices, the management of chest infections in mechanically ventilated patients remains a complex and challenging task.

BAL has become increasingly important as a diagnostic method for assessing suspected pulmonary infections, especially in mechanically ventilated patients. The procedure entails the introduction and subsequent removal of sterile saline solution into a particular lung segment, followed by the retrieval and analysis of the lavage fluid [6]. This method allows for the collection of samples from the lower respiratory tract, including the alveolar spaces, bronchi, and bronchioles, providing crucial insights into the microbial and cellular makeup of lung tissue.

The study objectives were to determine the occurrence of chest infections in mechanically ventilated patients, characterize the microbial profile of the organisms responsible for these infections, and assess the diagnostic and therapeutic efficacy of BAL in the management of patients with suspected chest infections. By addressing these key aspects, this article aims to provide a comprehensive understanding of chest infections in mechanically ventilated patients. Through this exploration, we seek to contribute to the optimization of clinical practices and the improvement of patient outcomes in critical care settings.

## Materials and methods

This prospective study was carried out in the Department of Anesthesia, Pain Management, and Critical Care, SN Medical College, Agra, India. The study was conducted from July 2022 to July 2023, and this protocol was approved by the institutional ethical committee at SN Medical College, Agra (IEC/2021/78). The inclusion criteria for the study included the patients admitted to the intensive care unit (ICU) with suspected chest infections or presenting with systemic signs of infection such as fever or changes in respiratory secretions, or radiological signs of infection, patients on mechanical ventilation and subjects with age more than 15 years and less than 65 years. The exclusion criteria included patients younger than 15 years or older than 65 years, those with anatomical abnormalities or obstructions in the bronchial tree, individuals with head or cervical injuries, patients who had already received broad-spectrum antibiotics, those not undergoing mechanical ventilation within the ICU, and patients with immunodeficiency conditions. The analysis involved a total of 38 subjects, ranging in age from 15 to 65 years, who were mechanically ventilated and underwent BAL procedure.

BAL procedures adhered to the guidelines outlined by the American Thoracic Society for Bronchoscopic Lavage [7]. Suspicion of pneumonia relied on clinical or radiological indicators or a simplified clinical pulmonary infectious score. To ensure patient safety and compliance, BAL was conducted under stringent aseptic precautions following the acquisition of informed written consent from participants. Routine investigations, including hemogram assessments, were conducted for all patients to obtain baseline clinical data. Furthermore, enteral nutrition was temporarily halted before and during the procedure to mitigate the risk of aspiration. Standardized protocols and precautions were implemented to uphold patient safety and optimize the efficacy of BAL procedures. A BAL was performed before any other bronchoscopic procedure. Trained consultants with prior experience conducted BAL procedures for all subjects. BAL fluid was collected using gentle suction. The procedure was monitored continuously, and vital signs were recorded at baseline, one hour, and 24 hours post-procedure. BAL samples were transported and processed immediately upon arrival at the laboratory. Microscopic investigation and quantitative culture techniques were employed for microbial analysis. Different staining methods were employed to detect and identify bacteria, fungi, and mycobacteria. Complications and adverse events were monitored and recorded. Equipment decontamination and disinfection were done before and after each patient's use. After the procedure, the equipment was cleaned meticulously, followed by chemical disinfection, adhering to both guidelines and manufacturer instructions.

In descriptive statistics, a mean with standard deviation was used to describe the data that was normally distributed. The categorical variables are expressed as frequency (%).

## Results

The study involved patients admitted to the ICU who were undergoing mechanical ventilation and were suspected to have chest infections based on clinical or radiological signs of infections. Table 1 shows the characteristics of the study participants. The study included 38 subjects, aged between 21 and 62 years, with a mean age of 40.52 years. Among the participants, 21 (55.2%) were male, while 17 (44.8%) were female.

**Table 1:** Characteristics of the study participants

Variables	n (%)
Age in years (mean±SD)	40.52±11.6
Age range in years (median)	21-62 (43)
Gender	
Male	21 (55.2%)
Female	17 (44.8%)
Co-morbidities	
Present	24 (63.2%)
Absent	14 (36.8%)

Table 2 describes the BAL samples' culture positivity. Out of the 38 bronchoalveolar lavage (BAL) samples collected, 30 exhibited positive results for microbial infection, accounting for a prevalence rate of 30 (78.94%). Additionally, five (13.15%) samples yielded inconclusive results, while three (7.9%) samples were negative and showed no growth of the microorganism. 

**Table 2:** Culture positivity of BAL samples BAL - broncho-alveolar lavage

Culture results	Number of samples	Percentage (%)
Positive culture (>10^4^ cfu/ml)	30	78.94
Inconclusive (<10^4 ^cfu/ml)	5	13.15
Negative (no growth)	3	7.89

Table 3 describes the microbial profile of the study sample. The microbiological analysis revealed a range of organisms involved in the infections. Escherichia coli emerged as the predominant organism, isolated from 14 (46.6%) out of the 30 positive samples. Following this, Klebsiella pneumonia (20%) and Acinetobacter baumannii (20%) were the next most frequently identified organisms. Meanwhile, Pseudomonas aeruginosa and Staphylococcus aureus were present in two (6.67%) of the samples, respectively. 

**Table 3:** Microbial profiles of the study samples

Organism	Number of samples	Percentage (%)
Escherichia coli	14	46.6
Klebsiella pneumonia	6	20
Acinetobacter baumannii	6	20
Pseudomonas aeruginosa	2	6.67
Staphylococcus aureus	2	6.67

Further investigation into antibiotic sensitivity revealed notable trends. Samples positive for Escherichia coli showed sensitivity primarily to meropenem, piperacillin+tazobactam, and cefoperazone+sulbactam, as well as complete resistance to fluoroquinolones. Also, samples that tested positive for Klebsiella pneumonia, Acinetobacter baumannii, and Pseudomonas aeruginosa had similar sensitivity profiles. They were sensitive to meropenem, piperacillin+tazobactam, and cefoperazone+sulbactam, but not to fluoroquinolones. On the other hand, samples positive for Staphylococcus aureus demonstrated resistance to piperacillin+tazobactam, cefoperazone+sulbactam, and the fluoroquinolone group but showed sensitivity to meropenem.

Table 4 describes the final outcomes of study participants who underwent the BAL procedure. After the BAL procedures, we categorized patient outcomes into three distinct groups. Eleven patients (28.94%) experienced full recovery, while 15 (39.47%) patients achieved partial recovery, necessitating further medical care and subsequent transfer to the wards. Unfortunately, 12 (31.57%) patients succumbed to their condition despite receiving adequate treatment and care within the ICU.

**Table 4:** Final outcomes of study participants

Final outcomes	n (%)
Full recovery	11 (28.94%)
Partial recovery	15 (39.47%)
Death	12 (31.57%)

## Discussion

The findings of this study shed light on the prevalence, microbial profiles, antibiotic sensitivity patterns, and outcomes of chest infections in mechanically ventilated patients, as assessed through BAL.

The observed prevalence rate of chest infections among mechanically ventilated patients, as determined by BAL, underscores the significance of this complication in critical care. With 78.94% of BAL samples showing positive results for microbial infection, our study underscores the high prevalence of respiratory infections within these patients. Chest infections are frequently acquired in ICU environments, with reported occurrence rates varying widely from 5% to 40%, influenced by diverse factors such as the setting and diagnostic criteria [8-11]. This finding highlights the crucial need for vigilant monitoring and proactive management approaches to reduce the risk of respiratory tract infections in mechanically ventilated patients. In a study led by Akthar et al., 26.08% of BAL samples showed positive results for bacterial isolates, which contrasts with the findings of other studies conducted by Velez et al. and Kottmann et al. where the positive yield was reported to be 51.6% and 55.8%, respectively [12-14].

From positive BAL samples, we identified Escherichia coli as the primary organism, followed by Klebsiella pneumoniae and Acinetobacter baumannii. These results are consistent with prior research indicating the predominance of gram-negative bacteria.

Our research also found that aerobic gram-negative bacilli were the most frequently isolated organisms. A similar pattern was observed in a recent study by Mishra et al., where they reported an incidence of 84.1% [15]. Additionally, several other studies have emphasized a notable predominance of gram-negative bacilli among respiratory pathogens [16-18]. The disproportionate distribution of patients with community-acquired and hospital-acquired infections, along with the rise of antibiotic resistance within hospital settings, may partially explain this prevalence of gram-negative bacteria. Few studies have indicated Klebsiella pneumoniae as the primary organism [19-21].

The antibiotic sensitivity profiles of the isolated pathogens provide valuable insights into antimicrobial stewardship and treatment strategies. Our results show that the organisms we found are very sensitive to carbapenems (like meropenem) and broad-spectrum β-lactam/β-lactamase inhibitor combinations (like piperacillin+tazobactam and cefoperazone+sulbactam). However, the widespread resistance to fluoroquinolones highlights the limited utility of this antibiotic class in the empirical management of chest infections in mechanically ventilated patients. These results emphasize the importance of judicious antibiotic use, susceptibility testing, and antimicrobial stewardship programs to combat antimicrobial resistance and optimize patient outcomes. Antimicrobial resistance in critical care settings poses a significant challenge due to the presence of multidrug-resistant pathogens like Acinetobacter baumannii. Understanding the microbial landscape of chest infections in mechanically ventilated patients is crucial for guiding empiric antibiotic therapy and implementing infection control measures effectively.

The outcomes of mechanically ventilated patients with chest infections are multifaceted and influenced by various factors, including the severity of the infection, comorbidities, and response to treatment. In our study, a substantial proportion of patients achieved partial recovery, necessitating further medical care and transfer to general wards. However, the mortality rate remains significant, with 31.57% of patients succumbing to their condition despite receiving adequate treatment and care within the ICU. These findings underscore the critical nature of chest infections in mechanically ventilated patients and highlight the need for timely diagnosis, aggressive management, and supportive care to improve patient outcomes.

This study's findings have several implications for clinical practice in critical care settings. Firstly, they underscore the importance of early detection and prompt treatment of chest infections in mechanically ventilated patients to prevent complications and improve outcomes. Secondly, the identification of prevalent pathogens and their antibiotic sensitivity profiles informs empiric antibiotic therapy and antimicrobial stewardship initiatives. Furthermore, the increased occurrence of multidrug-resistant organisms emphasizes the importance of implementing rigorous infection control measures and antimicrobial stewardship initiatives to reduce the spread of antimicrobial resistance in healthcare environments.

Limitations and future research

Despite its valuable contributions, this study has certain limitations that need to be acknowledged. Firstly, conducting the study at a single center may limit the generalizability of the findings to other healthcare settings. Secondly, the relatively small sample size could impact the statistical power and accuracy of the outcomes. Future research endeavors should aim to mitigate these limitations by conducting multicenter studies with larger sample sizes.

## Conclusions

In conclusion, our study emphasizes the significance of proactive monitoring, prompt diagnosis, and specific management needed to alleviate the impact of respiratory tract infections in critical care environments. Through improving diagnostic and treatment methods, healthcare professionals can improve patient outcomes and reduce the morbidity and mortality linked to chest infections in mechanically ventilated individuals.
